# The associations between proprotein convertase subtilisin/kexin type 9 E670G polymorphism and the risk of coronary artery disease and serum lipid levels: a meta-analysis

**DOI:** 10.1186/s12944-015-0154-7

**Published:** 2015-11-17

**Authors:** Gaojun Cai, Bifeng Zhang, Ganwei Shi, Weijin Weng, Chunyan Ma, Yanbin Song, Ji Zhang

**Affiliations:** Department of Cardiology, Wujin Hospital Affiliated to Jiangsu University, Changzhou, Jiangsu Province China; Department of Pathology and Molecular Medicine, McMaster University, Ontario, Canada

**Keywords:** Proprotein convertase subtilisin/kexin type 9, Dyslipidemia, Polymorphism, Gene, Coronary artery disease, Meta-analysis

## Abstract

**Background:**

Studies had investigated the associations between proprotein convertase subtilisin/kexin type 9 (*PCSK9*) E670G polymorphism and coronary artery disease (CAD) and lipid levels, but the results were controversial. Thus, we performed this meta-analysis to investigate the association between *PCSK9* E670G polymorphism and lipid levels and the susceptibility to CAD.

**Methods:**

All relevant articles according to the inclusion criteria were retrieved and included in the present meta-analysis. Odds ratios (ORs) with 95 % confidence interval (CI) were used to analyze the strength of the association between *PCSK9* E670G polymorphism and the susceptibility to CAD. At the same time, the pooled standardized mean difference (SMD) with 95 % CI was used for the meta-analysis of *PCSK9* E670G polymorphism and lipid levels. The publication bias was examined by using Begg’s funnel plots and Egger’s test.

**Results:**

A total of seventeen studies met the inclusion criteria. For CAD association, the pooled effects indicated that the G allele carriers had higher risk of CAD than non-carriers in dominant genetic model (OR:1.601, 95 % CI: 1.314–1.951, *P* < 0.001), as well as in allelic genetic model (OR: 1.546, 95 % CI: 1.301–1.838, *P* < 0.001). When the subgroup analysis stratified by ethnicity and HWE was performed, the positive result existed in most of the subgroups. For lipid levels association, the pooled effects indicated that the G allele carriers had higher TC and LDL-C levels than the non-carriers (for TC, SMD: 0.126, 95 % CI: 0.023–0.229, *P* = 0.016; for LDL-C, SMD: 0.170, 95 % CI: 0.053–0.287, *P* = 0.004, respectively). There was no difference in the levels of TG and HDL-C between the G carriers and the non-carriers in the whole population (SMD: 0.031, 95 % CI: −0.048–0.110, *P* = 0.440; SMD: −0.123, 95 % CI: −0.251–0.006, *P* = 0.061, respectively). When the studies were stratified by ethnicity and type of study, the G carriers had higher TC levels than the non-carriers (SMD: 0.126, 95 % CI: 0.014–0.238, *P* = 0.027) in the non-Asian subgroup. The similar results existed in cohort subgroup. The association between *PCSK9* E670G polymorphism and LDL-C levels was significant in all subgroups. Meanwhile, the G carriers had higher TG levels than the non-carriers (SMD: 0.113, 95 % CI: 0.012–0.214, *P* = 0.028) in the case–control subgroup. AG + GG genotypes had lower HDL-C levels than AA genotype in Asian subgroup (SMD: −0.224, 95 % CI: −0.423– −0.025, *P* = 0.027) and in case–control subgroup (SMD: −0.257, 95 % CI: −0.467–−0.048, *P* = 0.016).

**Conclusions:**

The present meta-analysis concluded that *PCSK9* E670G polymorphism was associated with CAD risk and lipid levels.

**Electronic supplementary material:**

The online version of this article (doi:10.1186/s12944-015-0154-7) contains supplementary material, which is available to authorized users.

## Background

Coronary artery disease (CAD) is the major manifestations of atherosclerotic process, which is expected to remain one of the leading causes of mortality until at least 2030 [1]. Epidemiological studies demonstrated that elevated serum low-density lipoprotein cholesterol (LDL-C) levels had consistently been shown to be the risk factors for the occurrence and development of CAD. In addition, genetic and other environmental factors are involved in the pathogenesis of CAD [2].

Proprotein convertase subtilisin/kexin type 9 (PCSK9), originally named neural apoptosis regulated convertase-1 (NARC-1), is the ninth member of the proprotein convertase family [3]. PCSK9 is mainly expressed in liver, kidney and intestine. The pro-PCSK9 undergoes auto-catalytic intramolecular cleavage at FAQ15↓2SIP residue in endoplasmic reticulum (ER) and Golgi apparatus. After the auto-catalytic procession, the mature and active PCSK9 protein is generated and secreted. By the catalytic domain, PCSK9 binds to the epidermal growth factor-like repeat A domain (EGF-A) of LDL receptor (LDLR) and enhances the degradation of LDLR, which increases the serum lipid levels and accelerates the procession of atherosclerosis. Recently, numerous studies revealed that PCSK9 was closely associated with lipid levels and the risk of CAD.

The coding gene for human PCSK9 protein is located on chromosome 1p32.3 and it encompasses 12 exons and encodes a 692 amino acid glycoprotein. It contains a signal peptide (1–30), a pro-domain (31–152) and a catalytic domain (153–451) followed by a cysteine-histidine rich domain (526–692). The pro-domain acts as an intramolecular chaperone, which ensures the catalytic domain to fold correctly in the ER.

The mutations of *PCSK9* were identified as the cause of a third monogenetic form of autosomal-dominant hypercholesterolemia (ADH), except LDLR and apolipoprotein B (apoB) genes [4]. Recently, there is an increasing interest in the role of the gene polymorphisms of *PCSK9* in the serum lipids homeostasis and the pathogenesis of CAD.

Up to date, a total of 163 mutations in *PCSK9* gene have been found, including 153 substitutions, eight insertions and two deletion mutations (www.ucl.ac.uk/ldlr/LOVDv.1.1.0/, the last update February 10, 2011). These mutations are divided into gain-of-function (GOF) and loss-of-function (LOF) mutations. LOF mutations in *PCSK9* gene increase the concentration of LDLR on the cell surface, which promotes the uptake of serum LDL-C and prevents the procession of CAD.GOF mutations enhance the degradation of LDLR, which reduces the uptakes of LDL-C and ultimately increases the circulating LDL-C levels. In addition, it may also regulate the production and secretion of apoB and enhance the levels of very low-density lipoprotein (VLDL) [5].

*PCSK9* E670G variant (rs505151), a common variant in exon 12, is located within the cysteine-rich C-terminal domain, which participates in the regulation of auto-catalysis [6]. The guanine is substituted for adenine at nucleotide 23,968, leading to a change from E to G at the position 670 of the PCSK9 protein, and this change may increase the affinity of PCSK9 for the LDLR. The frequency of 670G was varied greatly in different ethnic populations. Previous studies have revealed that E670G polymorphism was associated with high levels of total cholesterol (TC) and LDL-C and the risk of CAD [7, 8], but the results was inconsistent [9, 10]. For instance, Slimani A et al. found that the plasma TC and LDL-C levels were significantly higher in 670G carriers than in non-carriers and 670G increased the risk and the severity of CAD [8]. On the contrary, in a Chinese study, this variant was associated neither with elevated LDL-C levels, nor with the CAD susceptibility [10].

To enhance statistical power to produce a more precise result, we performed this meta-analysis based on available data aimed to derive a more precise association of the *PCSK9* gene polymorphism and lipid levels and the risk of CAD.

## Methods

### Studies selection

The meta-analysis followed the Perferred Reporting Items for Systematic Reviews and Meta-analysis (Additional file 1: PRISMA) criteria [11]. The electronic databases, such as PubMed, Foreign Medical Journal Service (FMJS) and Embase, were searched for relevant papers on the association between *PCSK9* E670G polymorphism and serum lipid levels and/or the risk of CAD. We also searched the Chinese electronic databases, such as Wanfang Data and China National Knowledge Infrastructure (CNKI) databases for Chinese literatures. The following keywords were used in the search: “PCSK”, “proprotein convertase subtilisin/kexin type 9”, “neural apoptosis regulated convertase-1”, “NARC-1”, “polymorphism”, “gene”, “rs505151”, “mutation”, “variant”, “lipid”, “dyslipidemia”, “coronary heart disease”,“myocardial infarction”, “coronary artery disease”, “ischemic heart disease”, “acute coronary syndrome”, “CAD” and “CHD”. The reference lists of the relevant papers that we identified were also checked.

### Inclusion and exclusion criteria

The eligible study must met the following criteria: 1) Case–control study or cohort study investigating the relationships between *PCSK9* E670G polymorphism and serum lipid levels and/or the risk of CAD; 2) For the lipid levels, the data were presented as mean ± standard deviations (SD) and the study had one of the serum lipid levels, at least including TC, triglyceride (TG), LDL-C or high density lipoprotein cholesterol (HDL-C); 3) For risk of CAD, the frequencies of genotypes in controls and CAD groups were clear.

Exclusion criteria: 1) The study was not conducted in human; 2) Family-based study. If there are several multiple publications from the same population, the most recent literature with the largest sample size was adopted.

### Data extraction

A special form was prepared for recording the available information. Two reviewers (Cai and Zhang) independently extracted the following data from each eligible study: first author, year of publication, country, ethnicity, genotype and allele distributions, geontyping method, lipid levels, type of study, Hardy-Weinberg equilibrium (HWE). The disagreements were resolved by consulting with the third author (Shi).

### Data analysis

The deviation from the HWE for the *PCSK9* E670G genotype distributions was assessed by Fisher’s exact test. The odds ratios (ORs) with 95 % confidence interval (CI) were applied to evaluate the strength of the association between the *PCSK9* E670G polymorphism and the susceptibility to CAD. Because the frequency of genotype GG in most of studies was low, the pooled ORs were only performed for allelic model (G vs. A) and dominant model (AG + GG vs. AA). The pooled standardized mean difference (SMD) with 95 % CI was used for the meta-analysis of *PCSK9* E670G polymorphism and lipid levels. We defined *PCSK9* 670G carriers as having the AG and GG genotypes. If the unit of the lipids is presented as mg/dl, we converted it to mmol/l. In several studies, the subjects were divided into different subgroups (e.g. male or female, drinker or non-drinker, CAD or ischemic stroke), so we treated each subpopulation as a separate comparison in our meta-analysis. Between-study heterogeneity was investigated and measured by using Cochran’s Q test. It was also detected by using the *I*^2^ statistic. If the between-study heterogeneity was significant (*I*^2^ > 50 %, *P* ≤ 0.05), a random-effect model (a Dersimonian-Laird method) was used to calculate the results. Otherwise, the fixed effect model (a Mantel-Haenszel method) was adopted [12].

Because of the significant heterogeneity among studies, we carried out sensitivity analysis while evaluating the results again by omitting one single study each time. We also performed the subgroup analysis stratified by ethnicity (“Asian” or “non-Asian”) and type of study (“case–control” or “cohort”) to explore the sources of heterogeneity. The publication bias of literature was examined by Begg’s funnel plots, which was verified by Egger’s linear regression test.

The STATA version 12.0 (StataCorp LP, College Station, Texas 77845 USA) was used for the meta-analysis.

## Results

### Characteristics of included studies

**Fig. 1:**
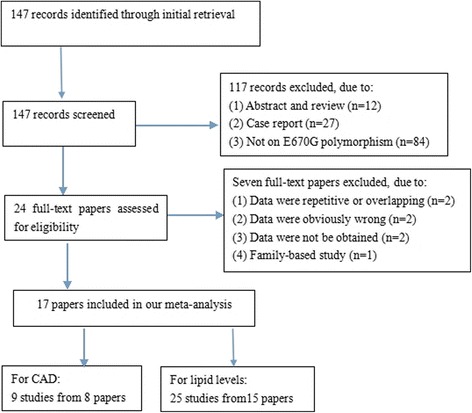
Flow diagram of article selection process for *PCSK9* E670G polymorphism and CAD and lipid levels

Through the initial retrieval, a total of 147 records were selected. 123 records were excluded according to abstract. Full-text articles were retrieved and seven literatures were excluded for the following reasons: (1) data were repetitive or overlapping (*n* = 2); (2) data were obviously wrong (*n* = 2); (3) data were not obtained (*n* = 2); (4) family-based study (*n* = 1). In the end, 17 literatures met all the inclusion criteria and are involved in the present meta-analysis [7, 8, 10, 13–26]. The flow diagram of the study selection process is presented in Fig 1.

All of the eligible studies included in this study had been approved by the Ethics Committee of their affiliations and our study was approved by the Ethics Committee of Wujin hospital, affiliated to Jiangsu University.

The characteristics of the included studies were listed in Tables 1 and 2. For CAD, nine comparisons from eight articles including 1517 cases and 1795 controls dealt with the association between *PCSK9* E670G polymorphism and risk of CAD. All of them were case–control studies and the minor allelic frequencies (MAF) varied greatly from 2.3 to 48.3 %. None of the distribution of genotypes in controls deviated from HWE, except Gao et al’s study. Among them, seven studies were performed among Asians, one among Caucasians and one among Africans, respectively. The genotyping method in most studies was PCR-RFLP, except for Mo et al’s study.

**Table 1:** Main characteristics of studies involved in this meta-analysis of PCSK9 E670G polymorphism and CAD risk

First author	Year	Country	Ethnicity	Mean age (years) (case/control)	Type of study	Sample size (case/control)	Cases			Controls			Genotyping method	MAF (control, %)	HWE (*P*)
							AA	AG	GG	AA	AG	GG			
Salazar LA [16]	2007	Chile	Caucasian	-/ -	C-C	110/108	105	5	0	103	5	0	PCR-RFLP	2.3	>0.05
Hsu LA [10]	2009	China	Asian	55.6 + 10.5/ 45.9 + 10.4	C-C	202/614	182	20	0	541	72	1	PCR-RFLP	6.0	>0.05
Zeng J [19]	2011	China	Asian	65.9 ± 10.2/ 57.0 ± 8.7	C-C	212/184	167	38	7	165	17	2	PCR-RFLP	5.7	>0.05
Meng YH [7]	2011	China	Asian	66.49 ± 9.92/ 64.34 ± 15.35	C-C	165/180	146	19	0	166	14	0	PCR-RFLP	3.9	>0.05
Slimani A [8]	2014	Tunisian	African	61[55–67]/ 49[45–55]	C-C	192/232	148	37	7	199	32	1	PCR-RFLP	7.3	>0.05
Zhang L [23]	2014	China	Asian	59.85 + 8.71/ 58.83 + 9.35	C-C	416/257	291	117	8	212	42	3	PCR-RFLP	15.3	>0.05
Mo YQ [26]	2015	China	Asian	56.4 + 11.7/ 54.7 + 10.2	C-C	100/100	87	13	0	92	8	0	DNA sequencing	4.0	>0.05
Gao Y ^a^ [25]	2015	China	Asian	-/-	C-C	60/60	19	21	20	20	22	18	PCR-RFLP	48.3	<0.05
Gao Y ^b^ [25]	2015	China	Asian	-/-	C-C	60/60	15	21	24	23	18	19	PCR-RFLP	46.7	<0.05

^a^Chinese Han population

^b^Mongols population

*C-C* case–control, *PCR-RFLP* polymerase chain reaction- restriction fragment length polymorphism, *MAF* minor allelic frequencies, *HWE* Hardy-Weinberg equilibrium

**Table 2:** Characteristics of individual studies included in the meta-analysis of PCSK9 E670G polymorphism and lipid levels

First author	Year	Country	Ethnicity	Type of study	Subpopulation	Genotype	Number (n)	TC (mmol/l)		TG (mmol/l)		HDL-C (mmol/l)		LDL-C (mmol/l)		Genotyping method
								M	SD	M	SD	M	SD	M	SD	
Chen SN 1 [13]	2005	American	Mixed	Cohort	LCAS population	AA	324	5.69	0.62	1.83	0.64	1.14	0.29	3.72	0.51	Allelic discrimination assays
						AG + GG	48	5.82	0.66	1.66	0.63	1.16	0.31	3.89	0.57	
Chen SN 2 [13]	2005	American	Caucasian	Cohort	TexGen population	AA	292	NA	NA	NA	NA	NA	NA	2.28	0.65	Allelic discrimination assays
						AG + GG	27	NA	NA	NA	NA	NA	NA	2.43	0.55	
Evans D 1 [14]	2006	Germany	Caucasian	Cohort	Male group	AA	190	6.05	1.4	1.39	0.45	1.16	0.31	4.29	1.34	PCR-RFLP
						AG + GG	26	6.57	1.48	1.55	0.44	1.2	0.29	4.65	1.37	
Evans D 2 [14]	2006	Germany	Caucasian	Cohort	Female group	AA	210	6.83	1.45	1.32	0.45	1.55	0.44	4.65	1.45	PCR-RFLP
						AG + GG	18	6.65	1.47	1.22	0.23	1.58	0.52	4.5	1.37	
Scartezini M [15]	2007	UK	Caucasian	Cohort	NPHSII men	AA	930	5.73	0.99	NA	NA	0.81	0.24	3.98	0.94	PCR-RFLP
						AG + GG	135	5.65	0.96	NA	NA	0.83	0.26	3.92	0.94	
Polisecki E 1 [17]	2008	American	Caucasian	Cohort	Male group	AA	2455	5.25	0.03	NA	NA	NA	NA	3.47	0.03	Taqman
						AG + GG	165	5.26	0.06	NA	NA	NA	NA	3.47	0.06	
Polisecki E 2 [17]	2008	American	Caucasian	Cohort	Female group	AA	2638	5.71	1.73	NA	NA	NA	NA	3.73	1.59	Taqman
						AG + GG	158	5.85	0.95	NA	NA	NA	NA	3.84	0.84	
Hsu LA 1 [10]	2009	Taiwan	Asian	C-C	CAD group	AA	541	5.14	0.96	1.59	1.3	1.42	0.37	3.02	0.85	PCR-RFLP
						AG	73	4.95	0.96	1.72	1.53	1.44	0.36	2.78	0.82	
Hsu LA 2 [10]	2009	Taiwan	Asian	C-C	Control group	AA	182	5.29	1.31	2.32	2.07	1.04	0.27	3.25	1.17	PCR-RFLP
						AG	20	5.28	0.74	2.48	1.4	1.07	0.33	3.41	0.6	
Norata GD 1 [18]	2010	Italy	Caucasian	Cohort	PLIC study	AA	1466	5.71	0.98	1.2	0.79	1.42	0.38	3.74	0.9	Taqman
						AG + GG	75	5.99	1.2	1.16	0.58	1.45	0.35	4.01	1.07	
Norata GD 2 [18]	2010	Italy	Caucasian	Cohort	Ventimiglia study	AA	728	4.76	0.99	NA	NA	NA	NA	3.02	0.86	Taqman
						AG + GG	48	4.89	0.98	NA	NA	NA	NA	3.23	0.87	
Zeng J [19]	2011	China	Asian	C-C	CAD group	AA	167	3.73	0.8	1.5	0.59	1.26	0.38	2.16	0.73	PCR-RFLP
						AG + GG	45	4.25	1.38	1.87	1.16	1.26	0.41	2.58	1.08	
Meng YH [7]	2011	China	Asian	C-C	CAD group	AA	146	4.41	0.72	0.99	0.62	1.48	0.51	2.22	0.63	PCR-RFLP
						AG	19	4.63	1.21	1.17	0.97	0.98	0.84	3.02	0.97	
Aung LHH 1 [21]	2013	China	Asian	Cohort	non-drinker group	AA	744	4.59	0.99	1.23	0.92	1.82	0.49	2.55	0.82	PCR-RFLP
						AG	41	5.01	1.25	1.09	0.78	1.86	0.51	2.68	0.86	
Aung LHH 2 [21]	2013	China	Asian	Cohort	Drinker group	AA	543	4.84	1.07	1.02	0.73	1.74	0.44	2.94	0.81	PCR-RFLP
						AG	24	4.55	0.64	1.1	0.58	1.63	0.5	2.69	0.44	
Mayne J [20]	2013	Canada	Caucasian	Cohort	African Canadian population	AA	192	5.56	1.14	1.59	0.77	1.2	0.4	3.64	1.01	PCR + full exonic sequencing
						AG	15	5.11	1.12	1.27	0.48	1.1	0.3	3.45	1.11	
Slimani A 1 [8]	2014	Tunisian	African	C-C	CAD group	AA	148	NA	NA	1.69	0.86	1	0.36	NA	NA	PCR-RFLP
						AG + GG	44	NA	NA	1.97	0.96	1.03	0.16	NA	NA	
Slimani A 2 [8]	2014	Tunisian	African	C-C	IS group	AA	90	NA	NA	1.58	0.75	1.35	0.4	NA	NA	PCR-RFLP
						AG + GG	24	NA	NA	1.47	0.58	1.04	0.19	NA	NA	
Anderson JM 1 [22]	2014	Brazil	Caucasian	C-C	HC group	AA	91	7.21	0.96	1.87	0.8	1.45	0.36	4.89	0.88	Taqman
						AG + GG	37	7.4	0.96	1.81	0.76	1.5	0.31	4.94	0.8	
Anderson JM 2 [22]	2014	Brazil	Caucasian	C-C	NL group	AA	131	4.47	0.47	0.91	0.32	1.53	0.36	2.53	0.47	Taqman
						AG + GG	40	4.5	0.49	0.95	0.3	1.47	0.26	2.59	0.47	
Zhang L 1 [23]	2014	China	Asian	C-C	CAD group	AA	291	4.07	1.16	1.82	0.79	1.37	0.16	2.29	0.77	PCR-RFLP
						AG + GG	125	4.49	1.31	1.87	1.09	1.31	0.25	2.5	0.74	
Zhang L 2 [23]	2014	China	Asian	C-C	Control group	AA	212	4.67	0.62	1.45	1.16	1.49	0.21	2.67	0.81	PCR-RFLP
						AG	45	4.53	0.33	1.37	0.88	1.4	0.21	2.63	0.8	
Jeenduang N 1 [24]	2015	Thai	Asian	Cohort	Male group	AA	132	5.54	1.3	1.46	0.84	1.32	0.35	3.66	1.13	PCR-RFLP
						AG	3	5.82	2.25	1.4	0.92	1.19	0.21	4	2.06	
Jeenduang N 2 [24]	2015	Thai	Asian	Cohort	Female group	AA	347	5.54	1.22	1.22	0.74	1.45	0.34	3.62	0.95	PCR-RFLP
						AG	13	5.99	0.94	1.01	0.35	1.49	0.23	4.04	0.94	
Mo YQ [26]	2015	China	Asian	C-C	CAD group	AA	87	4.48	0.81	0.97	0.58	1.49	0.47	2.12	0.72	DNA sequencing
						AG	13	4.56	0.97	1.05	0.89	0.97	0.73	3.01	0.83	

*C-C* case–control, *CAD* coronary artery disease, *IS* ischemic stroke, *NA* not available, *HC* hypercholesterolemics, *NL* normolipidemics, *UK* United Kingdom, *PCR-RFLP* polymerase chain reaction- restriction fragment length polymorphism

For lipid levels, ten papers separately provided data for more than one subpopulation (such as male and female groups, drinker and nondrinker groups, and so on), and we treated each subpopulation as a separate comparison. Therefore, 25 comparisons from 15 papers were used to evaluate the relationship between *PCSK9* E670G polymorphism and serum lipid levels, including 14,558 participants. Of these, 22 comparisons from 14 papers, 20 comparisons from 13 papers, 21 comparisons from 14 papers and 23 comparisons from 14 papers presented the data on TC, TG, HDL-C and LDL-C, respectively. 11 comparisons were conducted in Asians, 11 in Caucasians, and 3 in other ethnic populations. Genotype distributions in 19 populations or subpopulations were consistent with HWE. Seven papers were case–control studies and eight papers were cohort studies.

### Meta-analysis results

#### Association of the *PCSK9* E670G polymorphism with the risk of CAD

**Fig. 2:**
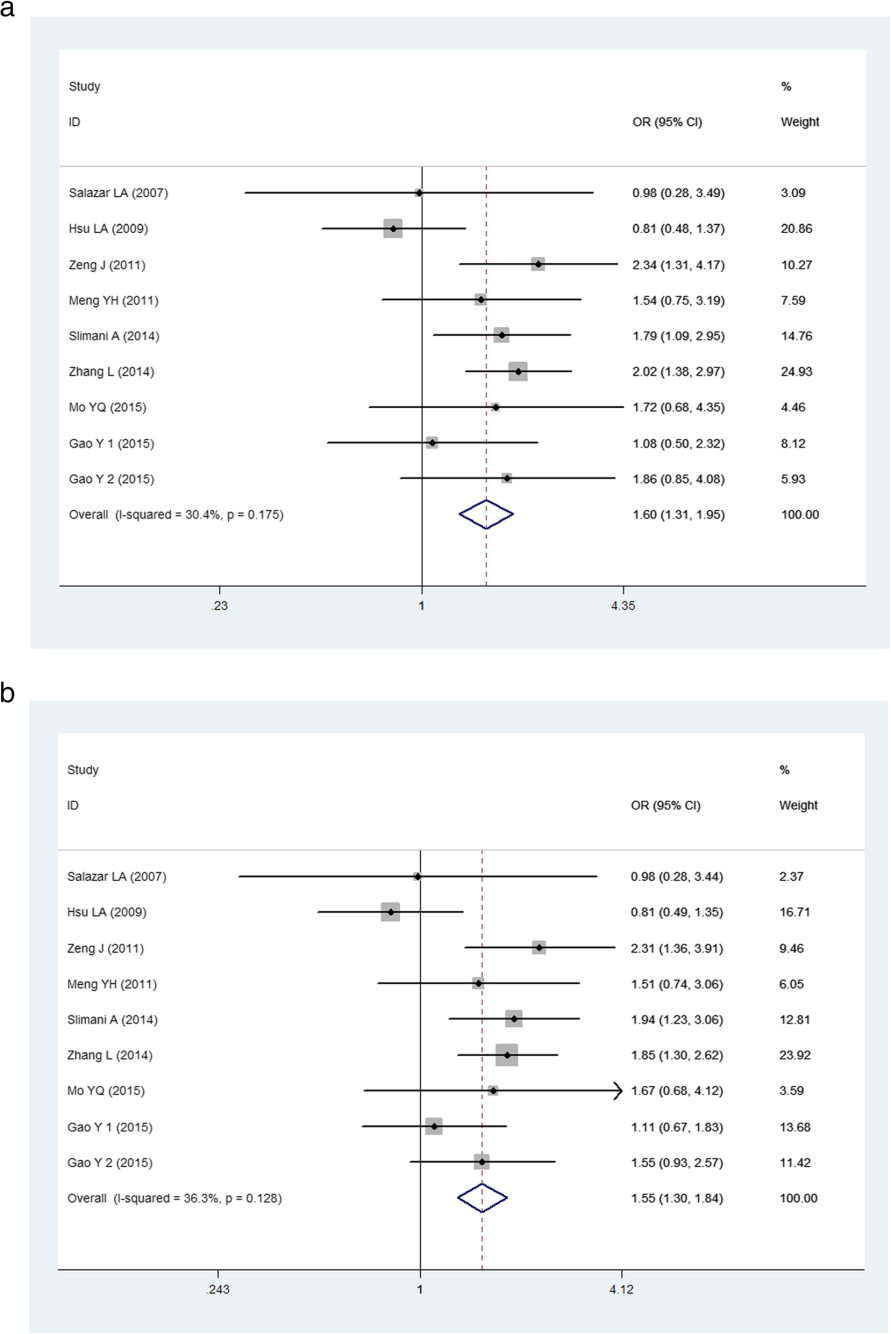
Forest plot of the association between *PCSK9* E670G polymorphism and the risk of CAD (**a**): dominant genetic model, AG + GG *vs* AA; (**b**): allelic genetic model, G *vs* A)

Due to the rare GG genotype, the dominant and allelic contrast genetic models were used to evaluate the association of the *PCSK9* E670G polymorphism with the risk of CAD. Because of absence of significant between-study heterogeneity, the fixed-effect model was applied. The pooled effects indicated that the G allele carriers had higher risk of CAD than non-carriers in dominant model (OR:1.601, 95 % CI: 1.314–1.951, *P* < 0.001), as well as in allelic model (OR: 1.546, 95 % CI: 1.301-1.838, *P* < 0.001) (Table 3, Fig. 2a, b).

**Table 3:** Summary of meta-analysis of association of PCSK9 E670G polymorphism and CAD risk

	*N*	*n* (CAD/controls)	Dominant model				Allelic model			
			OR (95 % CI)	P_OR_	*I* ^2^ (%)	P_Q_	OR (95 % CI)	P_OR_	*I* ^2^ (%)	P_Q_
All	9	1517/1795	1.601 (1.314–1.951)	<0.001	30.4	0.175	1.546 (1.301–1.838)	<0.001	36.3	0.128
Ethnicity										
Asians	7	1215/1455	1.590 (1.278–1.978)	<0.001	44.0	0.098	1.503 (1.244–1.816)	<0.001	45.5	0.088
non-Asians	2	302/340	1.652 (1.040–2.626)	0.034	0.0	0.386	1.788 (1.166–2.743)	0.008	0	0.318
HWE										
*P* > 0.05	7	1397/1675	1.633 (1.321–2.018)	<0.01	41.7	0.11	1.627 (1.335–1.983)	<0.001	43.6	0.100
*P* < 0.05	2	120/120	1.411 (0.819–2.430)	0.215	0	0.327	1.306 (0.912–1.869)	0.145	0	0.360

*OR* odds ratio, *CI* confidence interval, *P*_*OR*_ p values for odds ratio, *P*_*Q*_ p values for heterogeneity form Q-test, *HWE* Hardy-Weinberg equilibrium, *N* number of study, *n* number of individuals

Then the subgroup analyses stratified by ethnicity and HWE were performed. In the subgroup analyses stratified by ethnicity, significant associations were found among both Asians (for dominant model, OR: 1.590, 95 % CI: 1.278–1.978, *P* < 0.001; for allelic model, OR: 1.503, 95 % CI: 1.244–1.816, *P* < 0.001) and non-Asians (for dominant model, OR: 1.652, 95 % CI: 1.040–2.626, *P* = 0.034; for allelic model, OR: 1.788, 95 % CI: 1.166–2.743, *P* = 0.008). Subgroup analysis was also performed by HWE, significant association only existed in subgroup in HWE (for dominant model, OR: 1.633, 95 % CI: 1.321–2.018, *P* < 0.001; for allelic model, OR: 1.627, 95 % CI: 1.335–1.983, *P* < 0.001), suggesting that the results are credible.

### Association of the *PCSK9* E670G polymorphism with serum lipid levels

**Fig. 3:**
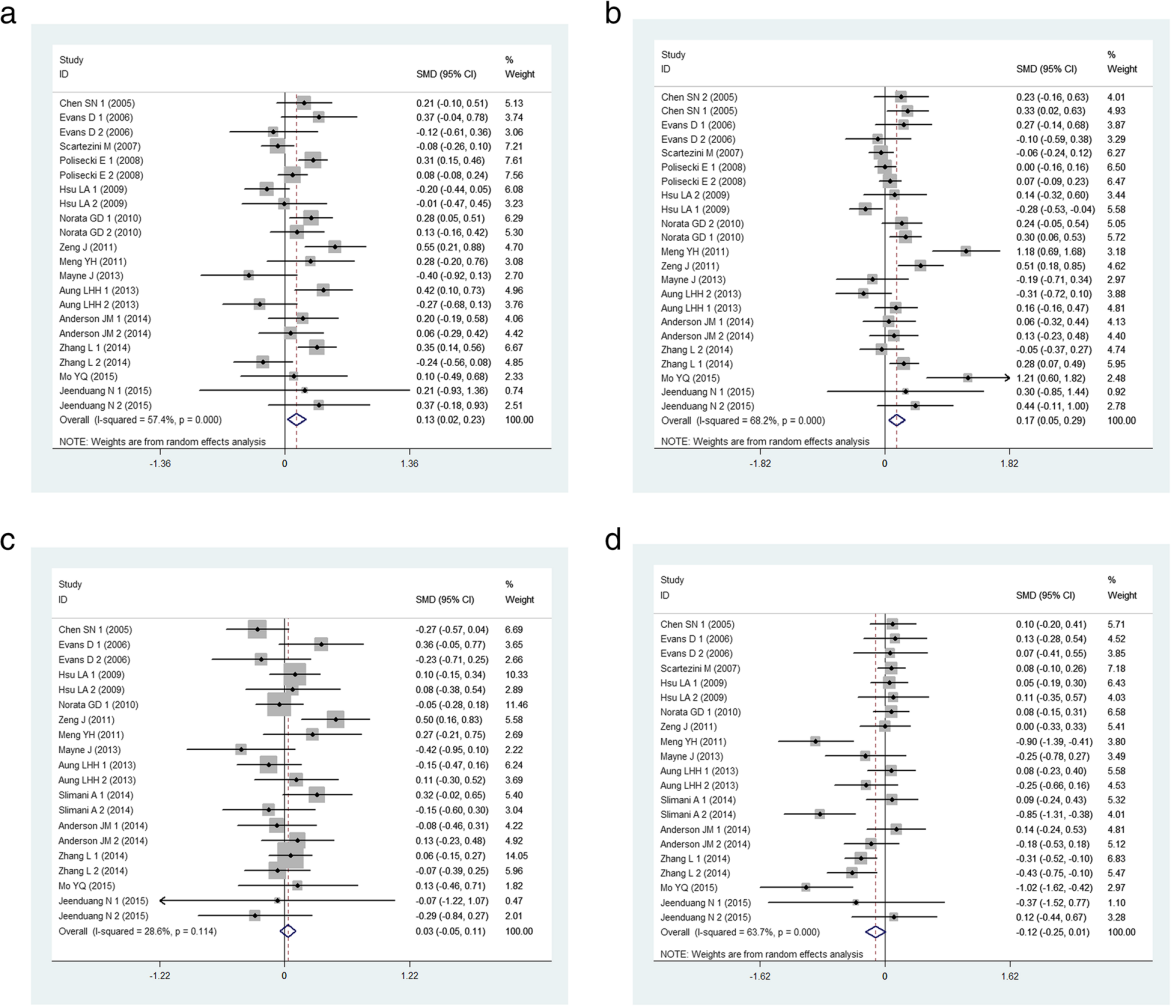
Forest plot of the associations between *PCSK9* E670G polymorphism and lipid levels. (**a**): for TC levels, random-effect model; (**b**): for LDL-C levels, random-effect model; (**c**): for TG levels, fixed effect model; (**d**): for HDL-C levels, random-effect model)

As shown in Table 4, in the whole population, the pooled effects indicated that the G allele carriers had higher TC and LDL-C levels than the non-carriers (for TC, SMD: 0.126, 95 % CI: 0.023–0.229, *P* = 0.016; for LDL-C, SMD: 0.170, 95 % CI: 0.053–0.287, *P* = 0.004, respectively) (Fig. 3a, b). There was no difference in the levels of TG and HDL-C between the G carriers and the non-carriers in the whole population (SMD: 0.031, 95 % CI: -0.048–0.110, *P* = 0.440; SMD: -0.123, 95 % CI: -0.251–0.006, *P* = 0.061, respectively) (Fig. 3c, d).

**Table 4:** Summary of meta-analysis of association of PCSK9 E670G polymorphism and lipid levels

	TC					TG						HDL-C				LDL-C				
Group and subgroups	N/n	SMD (95 % CI)	P	*I* ^2^ (%)	P_Q_	N/n	SMD (95 % CI)	P	*I* ^2^ (%)	P_Q_	N/n	SMD (95 % CI)	P	*I* ^2^ (%)	P_Q_	N/n	SMD (95 % CI)	P	*I* ^2^ (%)	P_Q_
All	22/13933	0.126 (0.023–0.229)	0.016	57.4	<0.001	20/6982	0.031 (−0.048–0.110)	0.440	28.6	0.114	21/8147	−0.123 (−0.251-0.006)	0.061	63.7	<0.001	23/14252	0.170 (0.053–0.287)	0.004	68.2	<0.001
Ethnicity																				
Asian	11/3813	0.133 (−0.067–0.334)	0.193	67.3	<0.001	11/3813	0.075 (−0.031–0.180)	0.164	12.6	0.325	11/3813	−0.224 (−0.423–−0.025)	0.027	66.5	0.001	11/3813	0.286 (0.021–0.551)	0.034	81.4	<0.001
non-Asian	11/10120	0.126 (0.014–0.238)	0.027	46.5	0.044	9/3169	−0.024 (−0.142–0.094)	0.688	41.5	0.090	104234	−0.017 (−0.165–0.132)	0.825	48.5	0.042	12/10439	0.097 (0.011–0.184)	0.027	18.8	0.259
Type of study																				
Cohort	13/11668	0.133 (0.010–0.256)	0.034	53.9	0.011	9/4411	−0.097 (−0.222–0.029)	0.132	14.4	0.31	10/5476	0.050 (−0.053–0.153)	0.342	0.0	0.883	14/11987	0.100 (0.004–0.197)	0.042	31.7	0.122
C-C	9/2265	0.119 (−0.077–0.315)	0.235	65.4	0.003	11/2571	0.113 (0.012–0.214)	0.028	7.1	0.376	11/2571	−0.257 (−0.467–−0.048)	0.016	74.3	<0.001	9/2265	0.307 (0.027–0.588)	0.031	83.3	<0.001

*C-C* case–control, *SMD* standardized mean difference, *CI* confidence interval, *P*_*OR*_ p values for odds ratio, *P*_*Q*_ p values for heterogeneity form Q-test, *TC* total cholesterol, *TG* triglyceride, *HDL-C* high density lipoprotein cholesterol, *LDL-C* low-density lipoprotein cholesterol, *N* number of study, *n* number of individuals

The subgroup analyses stratified by ethnicity and type of study were also performed. In the non-Asian subgroup, the G carriers had higher TC levels than the non-carriers (SMD: 0.126, 95 % CI: 0.014-0.238, *P* = 0.027). The similar results existed in cohort subgroup. The association between *PCSK9* E670G polymorphism and LDL-C levels was significant in all subgroups. Meanwhile, the G carriers had higher TG levels than the non-carriers (SMD: 0.113, 95 % CI: 0.012-0.214, *P* = 0.028) in the case–control subgroup. AG + GG genotypes had lower HDL-C levels than AA genotype in Asian subgroup (SMD: -0.224, 95 % CI: -0.423- -0.025, *P* = 0.027) and in case–control subgroup (SMD: -0.257, 95 % CI: -0.467--0.048, *P* = 0.016).

### Heterogeneity analysis

For CAD, the between-study heterogeneity was not significant (for dominant model, *I*^2^ = 30.4 %, *P* = 0.175; for allelic model, *I*^2^ = 36.3 %, *P* = 0.128). For lipid levels, there was significant heterogeneity among the total comparisons for TC, HDL-C and LDL-C (for TC, *I*^2^ = 57.4 %, *P*_heterogeneity_ < 0.001; for HDL-C, *I*^2^ = 63.7 %, *P*_heterogeneity_ < 0.001; for LDL-C, *I*^2^ = 68.2 %, *P*_heterogeneity_ < 0.001). In order to explore the possible sources of heterogeneity, subgroup analyses stratified by ethnicity (Asian or non-Asian) and type of study (cohort or case–control) were performed. But the between-study heterogeneity was not obviously reduced under the subgroup analyses.

### Sensitivity analysis

To evaluate the influence of single study on the whole result, the sensitivity analysis was carried out by calculating pooled estimates again when omitting a single study each time. The results suggested that no single study could influence the stability of the overall pooled estimates not only for CAD, but also for lipid levels (Additional file 2: Figure S1A-E).

### Publication bias

The Begg’s funnel plot was performed to assess the publication bias and no visual publication bias was found in all the comparisons, which was confirmed by Egger’s test (for CAD: *P* = 0.095; for TC: *P* = 0.143; for TG: *P* = 0.585; for HDL-C: *P* = 0.295; for LDL-C: *P* = 0.464, respectively) (Additional file 3: Figure S2A-E).

## Discussion

To our knowledge, the current meta-analysis was the first study to investigate the relationships between *PCSK9* gene E670G polymorphism and lipid levels and the risk of CAD. Our study concluded that *PCSK9* E670G polymorphism might be associated with lipid levels and the susceptibility to CAD.

A growing body of evidence indicated that PCSK9 might influence the serum lipid levels and the progression of atherosclerosis in vitro and in vivo. Through a post-transcriptional pathway, PCSK9 regulates the level of LDLR and then plays a major role in cholesterol homeostasis. In addition to the degradation of LDLR, PCSK9 may also have a potential effect on the apoB100, which may be another factor to atherosclerosis. The monoclonal antibodies of PCSK9 are going to the phase III clinical trials, which are expected to be the new hope for the patients with ADH.

In 2003, Abifadel et al. [27] firstly identified the mutation in the *PCSK9* gene. Since then, a large number of LOF and GOF mutations were reported. Among them, E670G, as the GOF mutation, is one of the most commonly investigated *PCSK9* gene polymorphisms. The association of *PCSK9* genetic polymorphism with the disorders of lipid profile and risk of CAD has been intensively studied, but the results are inconclusive [9, 13–15, 18, 26, 28]. Even in the same ethnic population, the results were also inconsistent [7, 10, 21]. The discrepancies of the associations between *PCSK9* E670G polymorphism and lipid levels and CAD among various populations may be due to the different characteristics of the study population, such as age, gender, ethnicity and/or environmental factors [29]. In addition, a small sample size may also be one cause. To diminish these influences, we retrieved all the relevant articles and analyzed the potential associations between *PCSK9* E670G polymorphism and lipid levels and susceptibility to CAD. The present meta-analysis showed that 670G carriers had higher levels of TC and LDL-C than non-carriers and higher risk of CAD in overall populations (SMD: 0.126, 95 % CI: 0.023-0.229, *P* = 0.016; SMD: 0.170, 95 % CI: 0.053-0.287, *P* = 0.004, respectively). The dominant model suggested that 670G carriers were at a 1.601-fold higher risk of CAD than non-carriers. In the whole population, there was no difference in the levels of TG and HDL-C between the 670G carriers and non-carriers.

For the lipid study, there was a significant between-study heterogeneity, which may affect the interpretation of the results. To explore the heterogeneity, the subgroup analyses stratified by ethnicity and type of study were performed. But, we still could not explain the source of heterogeneity entirely. In the subgroup analyses, we found that 670G carriers had high TC levels in non-Asian populations (SMD: 0.126, 95 % CI: 0.014-0.238, *P* = 0.027) and in cohort studies (SMD: 0.133, 95 % CI: 0.010-0.256, *P* = 0.034). The association between this mutation and LDL-C levels was significant in all subgroups. Interestingly, the 670G carriers had higher TG levels in case–control subgroup (SMD: 0.113, 95 % CI: 0.012-0.214, *P* = 0.028). Moreover, the 670G carriers had lower HDL-C levels in Asian population (SMD: -0.224, 95 % CI: -0.423- -0.025, *P* = 0.027) and case–control subgroup (SMD: -0.257, 95 % CI: -0.467- -0.048, *P* = 0.016). The sensitivity analysis found that the pooled effects did not change after excluding single study each time, which indicated that the results were stable.

As other meta-analyses, there were several inherent limitations in this study. Firstly and mainly, the between-study heterogeneity is significant, which was a potential problem that may affect the interpretation of the results. As we all know, the between-study heterogeneity may be influenced by age, sex, sample size, type of study, and so on. Although the subgroup analyses were performed, the heterogeneity was not explained entirely. Secondly, the relationships between this SNP and CAD risk and lipid levels did not consider the confounding factors, such as age, sex, smoking, drinking and other lifestyle factors. In the study conducted by Evans et al, E670G polymorphism was associated with increased LDL-C levels in man but not in women. Thirdly, the number of studies included in the meta-analysis was small for CAD risk. Only one study was conducted in Caucasian population and one study was in African population. Fourthly, although the Egger’s tests indicated no remarkable publication bias in our meta-analysis, the inevitable publication bias may remain in the results because the papers having negative result were probably more difficult to be accepted for publication. Finally, the present meta-analysis was not able to assess gene-gene and gene-environment interactions.

## Conclusions

Despite the limitations, the present meta-analysis concluded that *PCSK9* E670G polymorphism was associated with CAD risk and lipid levels.

## Additional files

Additional file 1:
**Checklist S1 PRISMA Checklist.** (DOC 54 kb) Additional file 2: Figure S1. Analysis of influence of individual study on the pooled estimate in dominant model for CAD risk and lipid levels. Open circle indicates that the pooled odds ratio, given named study is omitted. Horizontal lines represent the 95 % confidence intervals. (S1A: for CAD; S1B: for TC levels; S1C: for TG levels; S1D: for HDL-C levels; S1E: for LDL-C levels). (ZIP 385 kb) Additional file 3: Figure S2. Funnel plot for study of the associations between *PCSK9* E670G polymorphism and the risk of CAD and lipid levels. Each point represents a separate study for the indicated association. Logor (y axis): the log of OR; s. e. of logor (x axis): the standard error of log (OR). (S2A: for CAD; S2B: for TC levels; S2C: for TG levels; S2D: for HDL-C levels; S2E: for LDL-C levels). (ZIP 108 kb)
