# Supplementary figures and images for: The associations between proprotein convertase subtilisin/kexin type 9 E670G polymorphism and the risk of coronary artery disease and serum lipid levels: a meta-analysis
Source: Lipids Health Dis. 2015 Nov 17;14:149. doi: 10.1186/s12944-015-0154-7 (PMC4650262; doi:10.1186/s12944-015-0154-7)

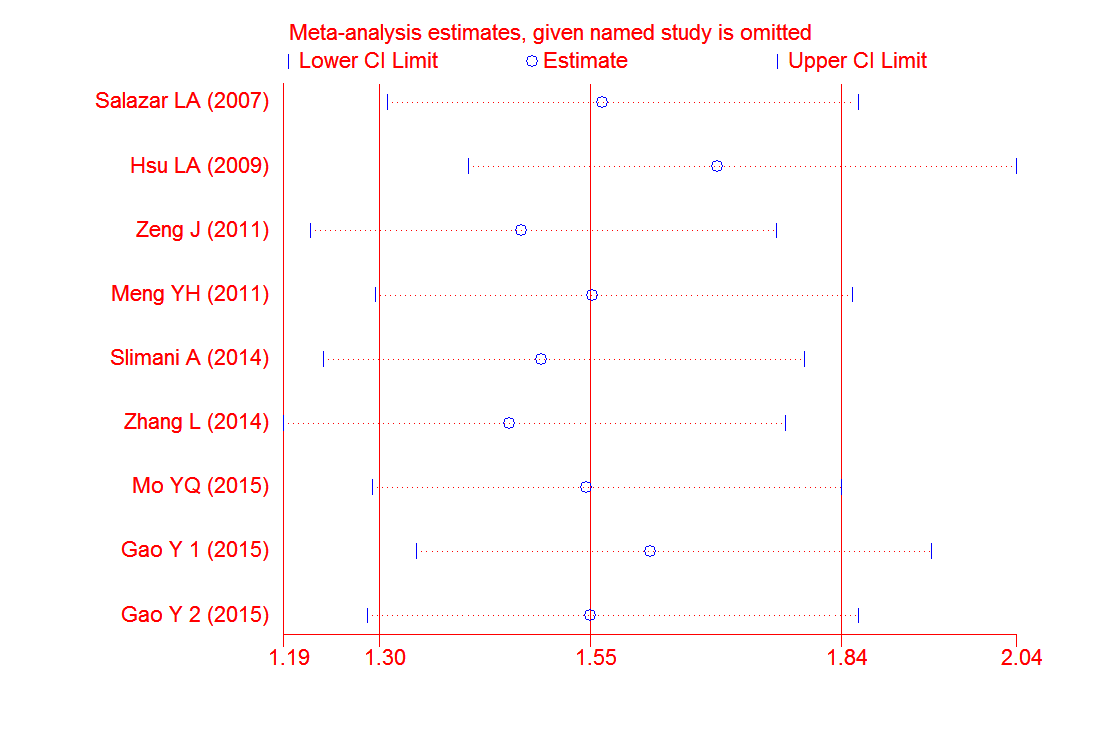

Supplement: Additional file 2: Figure S1. — Analysis of influence of individual study on the pooled estimate in dominant model for CAD risk and lipid levels. Open circle indicates that the pooled odds ratio, given named study is omitted. Horizontal lines represent the 95 % confidence intervals. (S1A: for CAD; S1B: for TC levels; S1C: for TG levels; S1D: for HDL-C levels; S1E: for LDL-C levels). (ZIP 385 kb) [file 12944_2015_154_MOESM2_ESM.zip › add1/Figure S1A.tif]

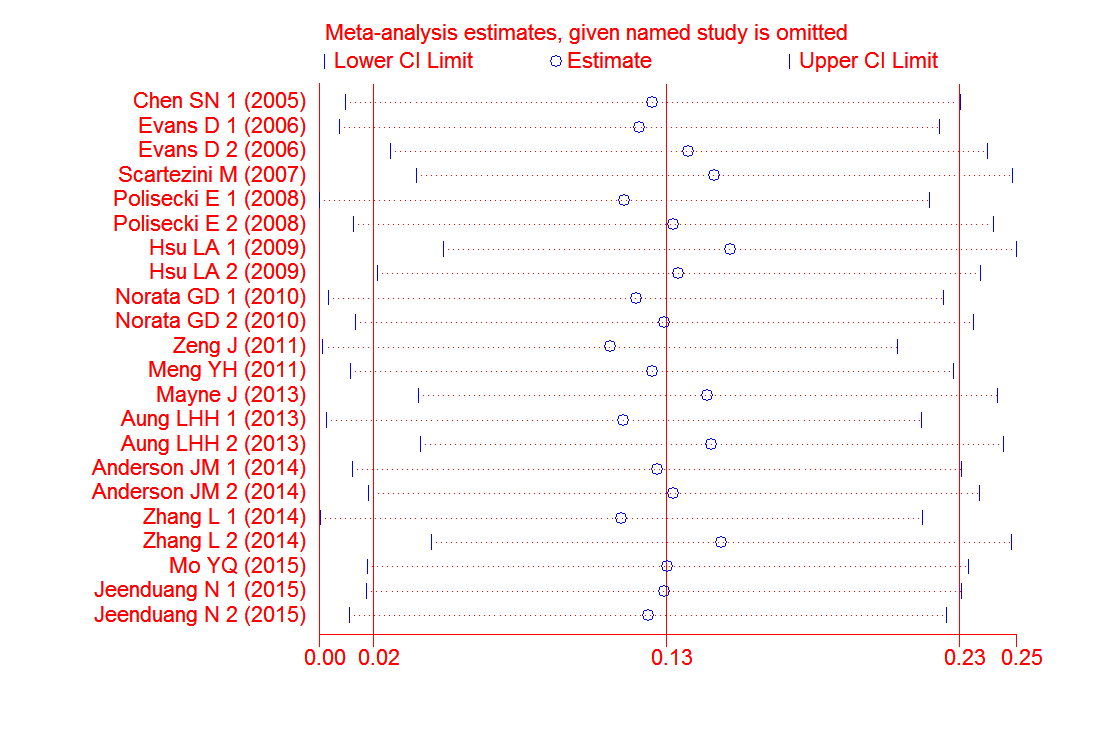

Supplement: Additional file 2: Figure S1. — Analysis of influence of individual study on the pooled estimate in dominant model for CAD risk and lipid levels. Open circle indicates that the pooled odds ratio, given named study is omitted. Horizontal lines represent the 95 % confidence intervals. (S1A: for CAD; S1B: for TC levels; S1C: for TG levels; S1D: for HDL-C levels; S1E: for LDL-C levels). (ZIP 385 kb) [file 12944_2015_154_MOESM2_ESM.zip › add1/Figure S1B.tif]

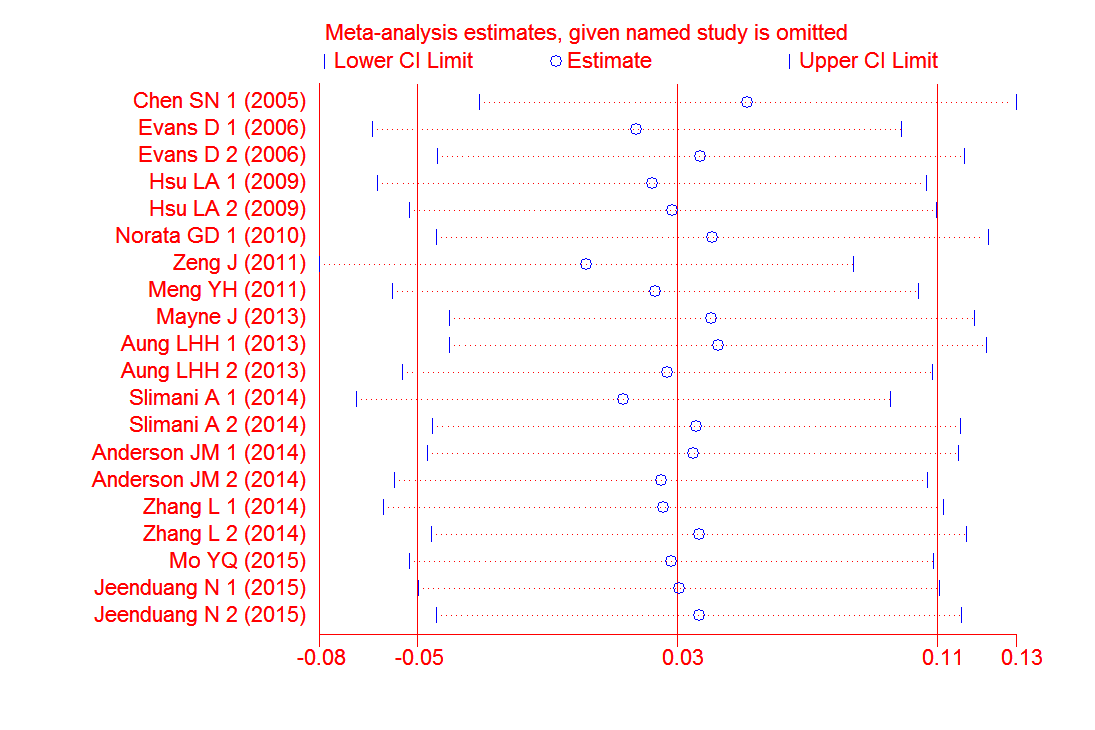

Supplement: Additional file 2: Figure S1. — Analysis of influence of individual study on the pooled estimate in dominant model for CAD risk and lipid levels. Open circle indicates that the pooled odds ratio, given named study is omitted. Horizontal lines represent the 95 % confidence intervals. (S1A: for CAD; S1B: for TC levels; S1C: for TG levels; S1D: for HDL-C levels; S1E: for LDL-C levels). (ZIP 385 kb) [file 12944_2015_154_MOESM2_ESM.zip › add1/Figure S1C.tif]

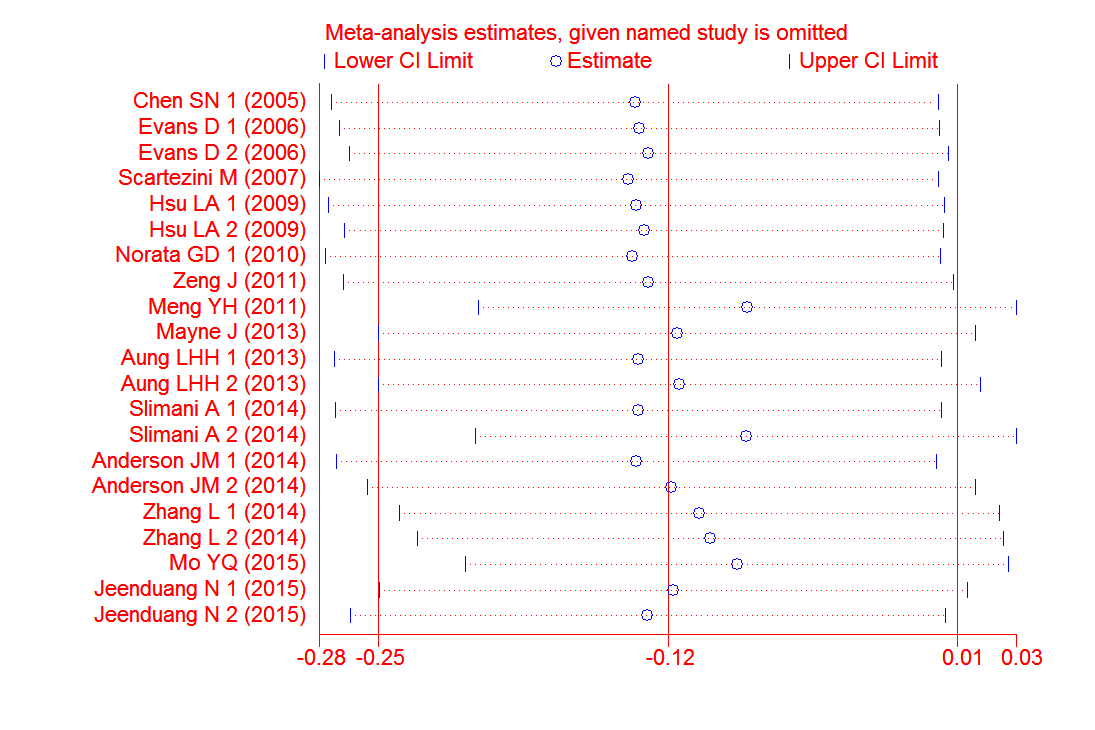

Supplement: Additional file 2: Figure S1. — Analysis of influence of individual study on the pooled estimate in dominant model for CAD risk and lipid levels. Open circle indicates that the pooled odds ratio, given named study is omitted. Horizontal lines represent the 95 % confidence intervals. (S1A: for CAD; S1B: for TC levels; S1C: for TG levels; S1D: for HDL-C levels; S1E: for LDL-C levels). (ZIP 385 kb) [file 12944_2015_154_MOESM2_ESM.zip › add1/Figure S1D.tif]

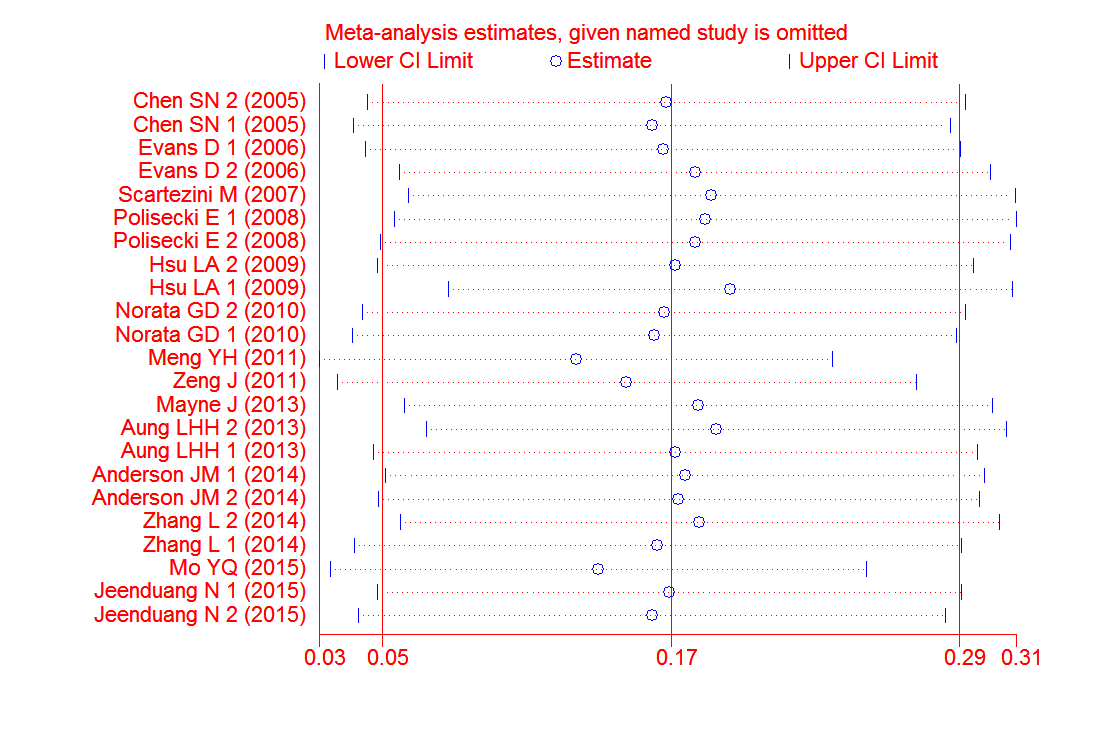

Supplement: Additional file 2: Figure S1. — Analysis of influence of individual study on the pooled estimate in dominant model for CAD risk and lipid levels. Open circle indicates that the pooled odds ratio, given named study is omitted. Horizontal lines represent the 95 % confidence intervals. (S1A: for CAD; S1B: for TC levels; S1C: for TG levels; S1D: for HDL-C levels; S1E: for LDL-C levels). (ZIP 385 kb) [file 12944_2015_154_MOESM2_ESM.zip › add1/Figure S1E.tif]

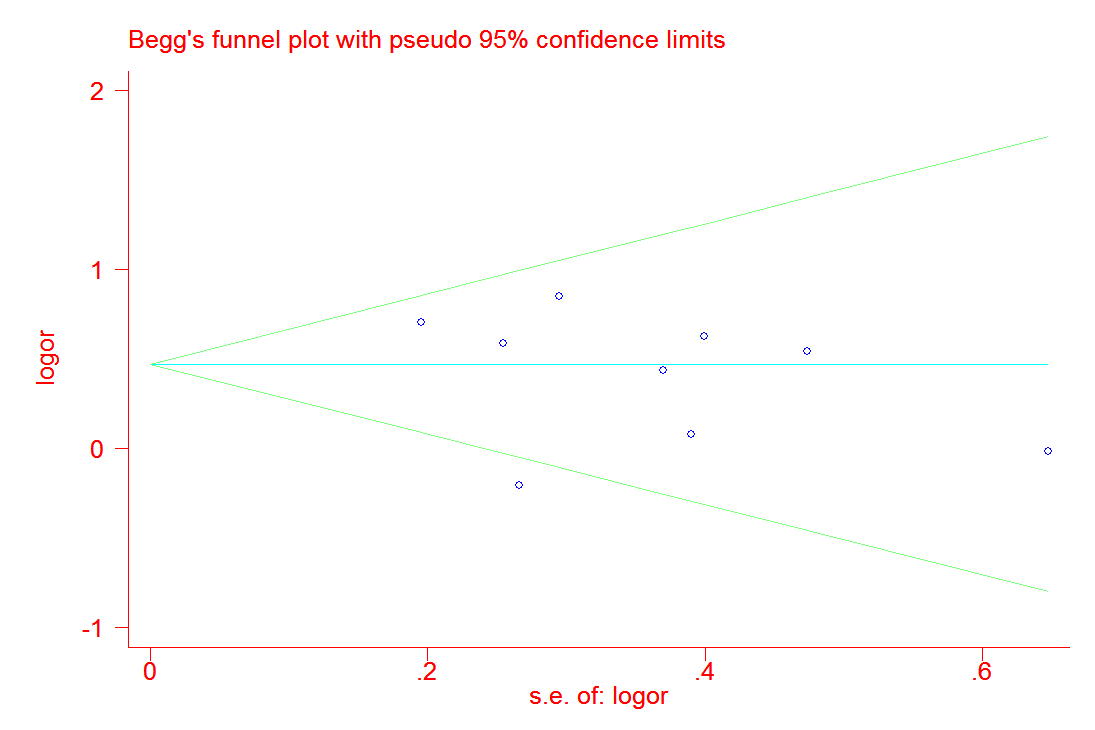

Supplement: Additional file 3: Figure S2. — Funnel plot for study of the associations between PCSK9 E670G polymorphism and the risk of CAD and lipid levels. Each point represents a separate study for the indicated association. Logor (y axis): the log of OR; s. e. of logor (x axis): the standard error of log (OR). (S2A: for CAD; S2B: for TC levels; S2C: for TG levels; S2D: for HDL-C levels; S2E: for LDL-C levels). (ZIP 108 kb) [file 12944_2015_154_MOESM3_ESM.zip › add2/Figure S2A.tif]

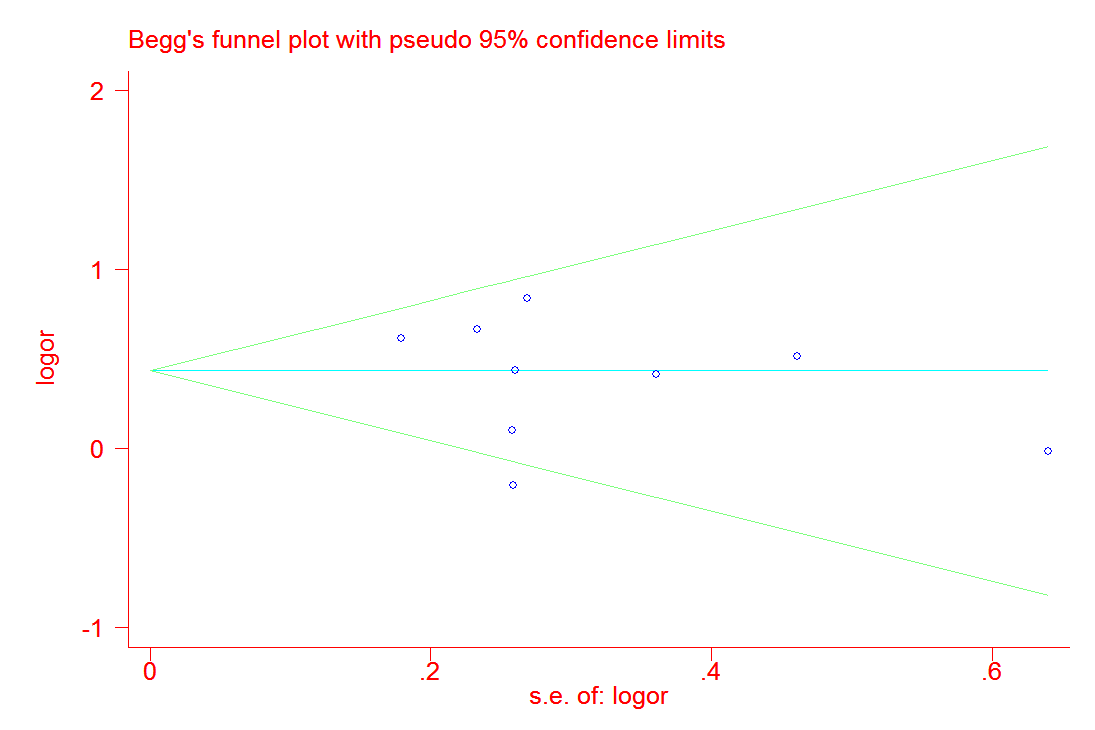

Supplement: Additional file 3: Figure S2. — Funnel plot for study of the associations between PCSK9 E670G polymorphism and the risk of CAD and lipid levels. Each point represents a separate study for the indicated association. Logor (y axis): the log of OR; s. e. of logor (x axis): the standard error of log (OR). (S2A: for CAD; S2B: for TC levels; S2C: for TG levels; S2D: for HDL-C levels; S2E: for LDL-C levels). (ZIP 108 kb) [file 12944_2015_154_MOESM3_ESM.zip › add2/Figure S2B.tif]

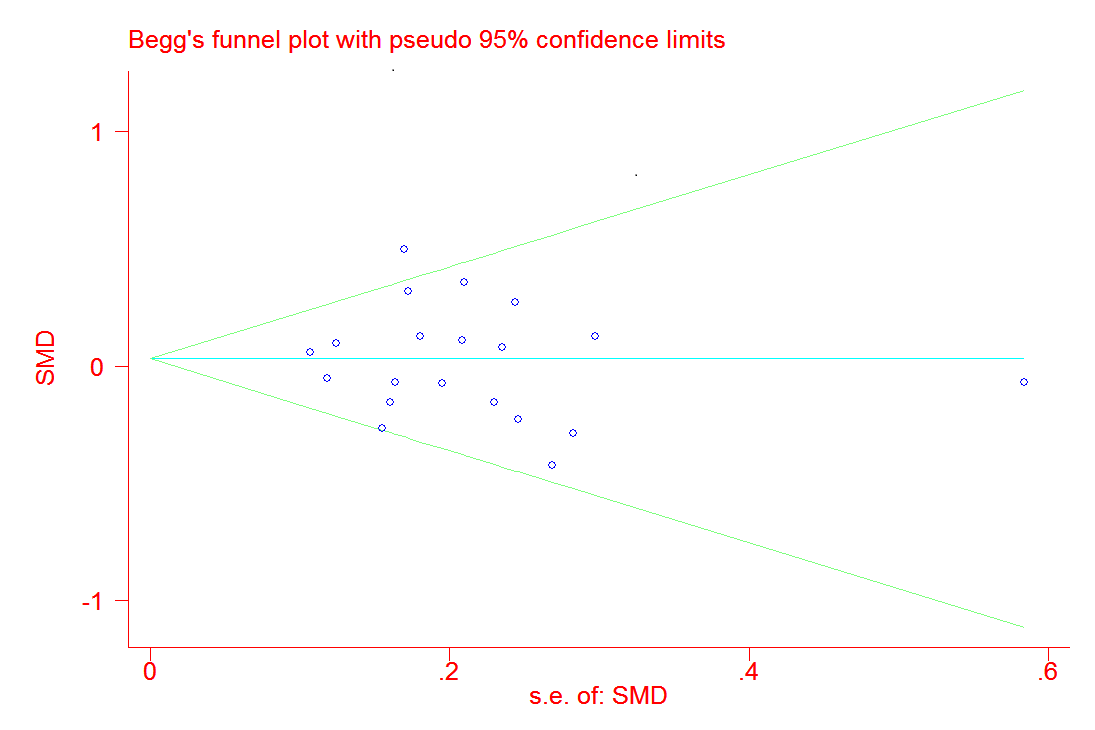

Supplement: Additional file 3: Figure S2. — Funnel plot for study of the associations between PCSK9 E670G polymorphism and the risk of CAD and lipid levels. Each point represents a separate study for the indicated association. Logor (y axis): the log of OR; s. e. of logor (x axis): the standard error of log (OR). (S2A: for CAD; S2B: for TC levels; S2C: for TG levels; S2D: for HDL-C levels; S2E: for LDL-C levels). (ZIP 108 kb) [file 12944_2015_154_MOESM3_ESM.zip › add2/Figure S2C.tif]

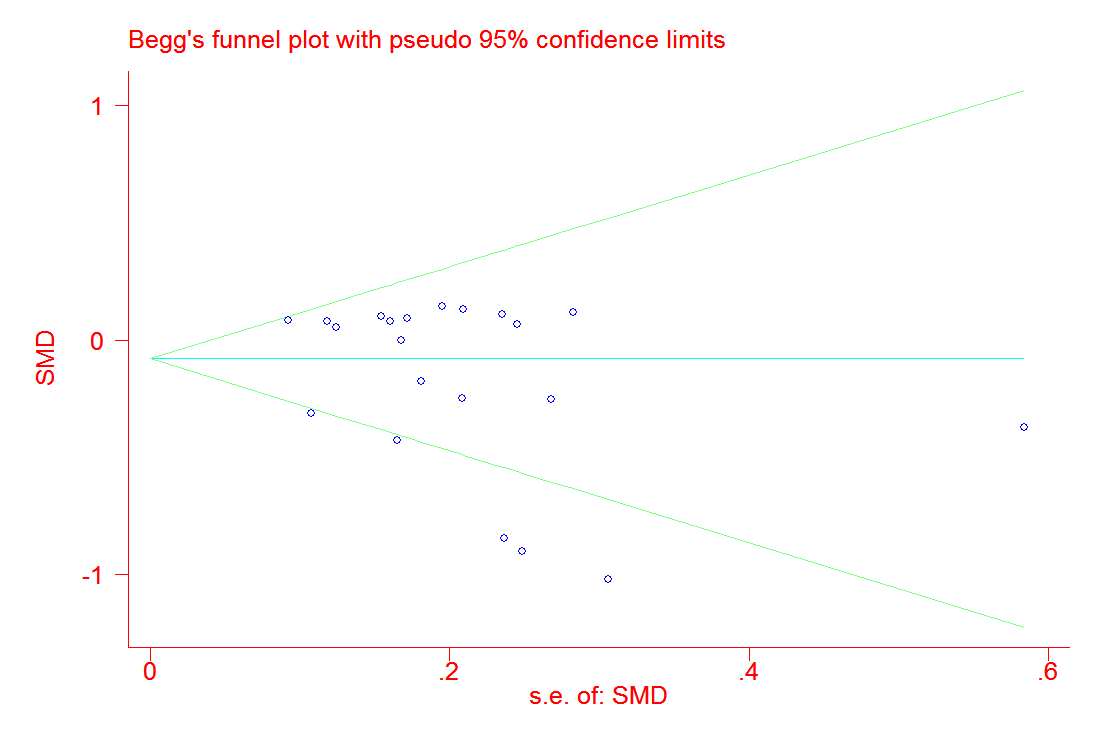

Supplement: Additional file 3: Figure S2. — Funnel plot for study of the associations between PCSK9 E670G polymorphism and the risk of CAD and lipid levels. Each point represents a separate study for the indicated association. Logor (y axis): the log of OR; s. e. of logor (x axis): the standard error of log (OR). (S2A: for CAD; S2B: for TC levels; S2C: for TG levels; S2D: for HDL-C levels; S2E: for LDL-C levels). (ZIP 108 kb) [file 12944_2015_154_MOESM3_ESM.zip › add2/Figure S2D.tif]

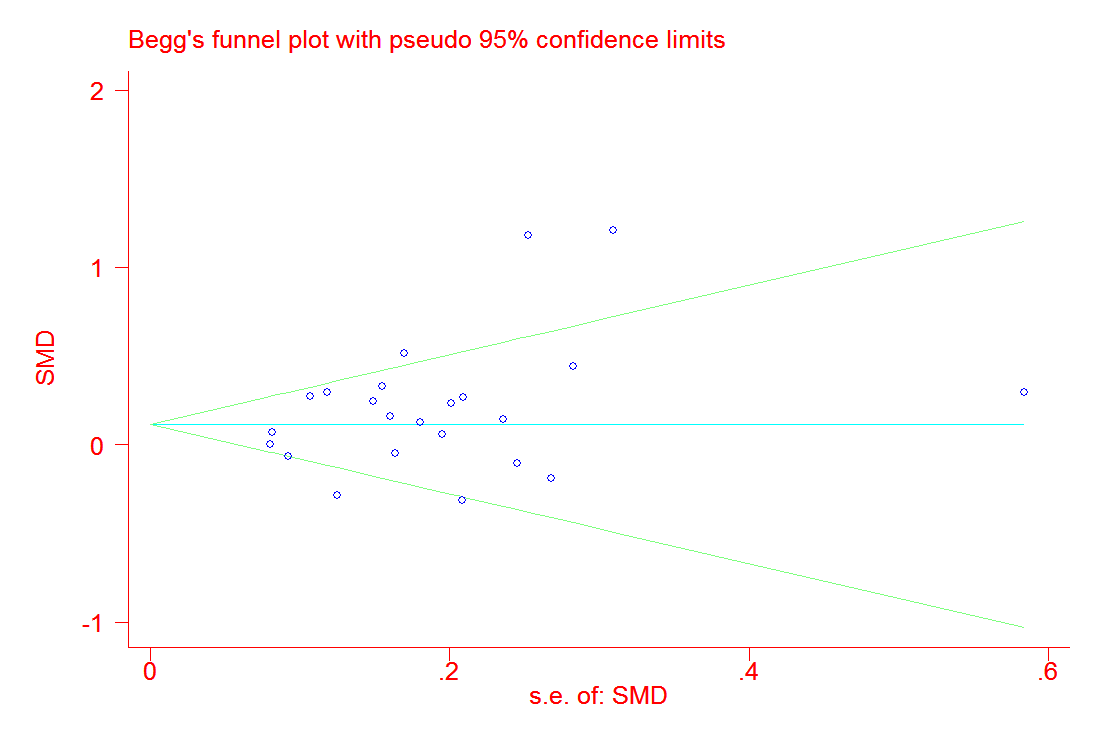

Supplement: Additional file 3: Figure S2. — Funnel plot for study of the associations between PCSK9 E670G polymorphism and the risk of CAD and lipid levels. Each point represents a separate study for the indicated association. Logor (y axis): the log of OR; s. e. of logor (x axis): the standard error of log (OR). (S2A: for CAD; S2B: for TC levels; S2C: for TG levels; S2D: for HDL-C levels; S2E: for LDL-C levels). (ZIP 108 kb) [file 12944_2015_154_MOESM3_ESM.zip › add2/Figure S2E.tif]
